# Periodic GFN1-xTB Tight Binding: A Generalized Ewald Partitioning Scheme for the Klopman–Ohno Function

**DOI:** 10.1021/acs.jctc.4c01234

**Published:** 2025-02-05

**Authors:** Alexander Buccheri, Rui Li, J. Emiliano Deustua, S. Mohamad Moosavi, Peter J. Bygrave, Frederick R. Manby

**Affiliations:** † School of Chemistry, University of Bristol, Cantocks Close, Bristol BS8 1TS, United Kingdom; ‡ Division of Chemistry and Chemical Engineering, California Institute of Technology, Pasadena, California 91125, United States; § Chemical Engineering and Applied Chemistry, University of Toronto, Toronto, Ontario M5S 3E5, Canada; ⊥ Department of Physics, Max Planck Institute for the Structure and Dynamics of Matter, Luruper Ch 149, 22761 Hamburg, Germany

## Abstract

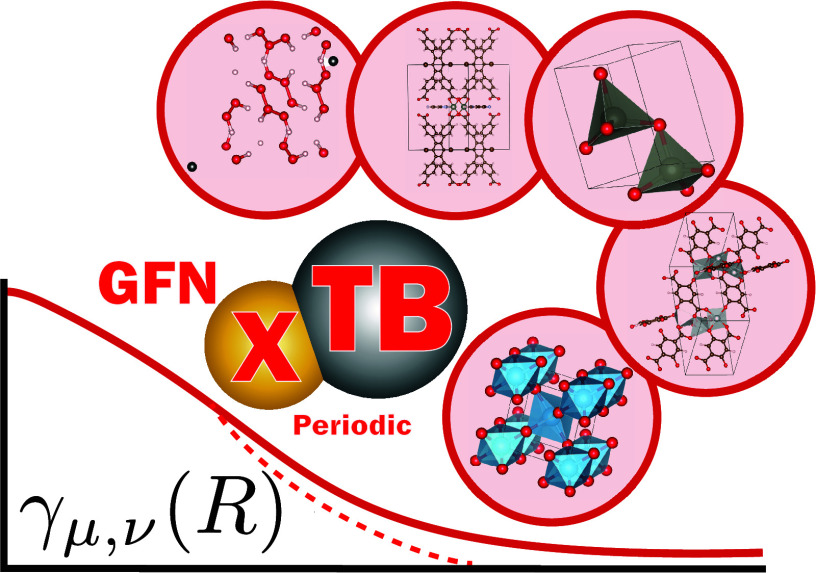

A novel formulation is presented for the treatment of electrostatics in the periodic GFN1-xTB tight-binding model. Periodic GFN1-xTB is hindered by the functional form of the second-order electrostatics, which only recovers Coulombic behavior at large interatomic distances and lacks a closed-form solution for its Fourier transform. We address this by introducing a binomial expansion of the Klopman–Ohno function to partition short- and long-range interactions, enabling the use of a generalized Ewald summation for the solution of the electrostatic energy. This approach is general and is applicable to any damped potential of the form |*R*^*n*^ + *c*|^–*m*^. Benchmarks on the X23 molecular crystal dataset and a range of prototypical bulk semiconductors demonstrate that this systematic treatment of the electrostatics eliminates unphysical behavior in the equation of state curves. In the bulk systems studied, we observe a mean absolute error in total energy of 35 meV/atom, comparable to the machine-learned universal force field, M3GNet, and sufficiently precise for structure relaxation. These results highlight the promising potential of GFN1-xTB as a universal tight-binding parametrization.

## 1 Introduction

Semiempirical tight-binding (TB) has long been a popular model with which to describe electronic structure in chemistry^1,2^ and physics^3−5^ because it provides an intuitive, real-space representation of valence molecular orbitals using a minimal, local orbital basis, and can be up to 3 orders of magnitude faster than DFT. In the past two decades, there has been a renewed interest in semiempirical TB methods within both quantum chemistry and materials science for simulating nanoscale systems that contain thousands of atoms.^6−8^

GFN1-xTB is a novel tight-binding model designed to yield reasonable geometries, vibrational frequencies, and noncovalent interactions for molecular systems.^9^ A tight-binding model is defined by a minimal orbital basis, and a set of approximations applied to the full DFT total energy. Self-consistency is achieved through a Taylor expansion of the total energy with respect to the fluctuation in charge density.^10^ The approximations in GFN1-xTB are broadly consistent with those made in DFTB3:^11^ A minimal, nonorthogonal atomic orbital basis (with the exception of higher-row elements, which include d-polarization functions), expansion of the total energy to third order in the charge density, a point-charge treatment of electrostatics, and a two-center approximation that restricts interactions to diatomic pairs. One crucial distinction is that the model is formulated in terms of elemental, rather than pairwise parameters. This trade-off sacrifices a degree of accuracy in order to significantly extend the scope of systems that can be simulated, and also facilitates the use of a simple functional form for the repulsive energy. By adopting this approach, the GFN-xTB molecular parametrization extends to all elements in the periodic table up to radon (*Z* = 86). Notably, GFN1-xTB is not the first tight-binding model to employ an element-wise parametrization scheme. Heine et al.^12^ previously developed a DFTB parametrization for band energies, covering most of the periodic table. This work was later expanded to include repulsive potentials for a limited set of light elements with atomic numbers *Z* ≤ 19 (and bromine).^13^ More recently, Cui et al.^14^ introduced an open-source DFTB parametrization for all elements up to radon, which also utilizes an element-wise approximation to the DFTB repulsive energy.

GFN1-xTB has demonstrated robust performance across a wide range of molecules in complex chemical environments. Notably, it has shown to be accurate in reproducing electron ionization mass spectra of organic, main-group, and transition metal complexes,^15^ electrochemical redux potentials of organometallic molecules in solvents,^16^ and infrared spectra of organic molecules in the gas phase.^17^ Furthermore, GFN1-xTB has found a broad range of applications including simulating optoelectronic properties,^18−20^ characterizing molecular structures,^21,22^ high-throughput discovery,^23−27^ molecular dynamics simulations,^28^ QM/MM embedding,^29−31^ and machine learning of molecular potential energy surfaces.^32^ Given the model’s impressive performance for molecular systems, and the vast chemical space potentially accessible through its element-wise parametrization scheme, there is a strong motivation to use GFN1-xTB for the treatment of periodic, extended and bulk systems.

GFN1-xTB has been formally extended to periodic boundary conditions,^33^ and is implemented in several electronic structure packages.^34−37^ However, in its current form, the second-order γ-potential cannot be screened and Fourier-transformed using the standard Ewald treatment of electrostatics. Existing implementations address this limitation by smoothly forcing components of the potential to zero at an arbitrary cutoff. In this paper, we outline the extension of GFN1-xTB with periodic boundary conditions, discuss the issues associated with the Klopman–Ohno (KO) functional form of the γ-potential, and introduce a partitioning scheme that enables the use of a generalized Ewald summation for its solution. The scheme is general, and can be applied to any damped potential of the form |*R*^*n*^ + *c*|^–*m*^. We present benchmarks of our implementation on a range of molecular and bulk crystals. For validation, we compare our results to plane wave DFT calculations (GGA-PBESOL^38^) performed with Quantum Espresso.^39,40^ To demonstrate the effect of our electrostatics treatment, we also compare our results to GFN1-xTB calculations that smoothly force the γ-potential to zero. We refer to these as GFN1-xTB(s) calculations, where (s) denotes smooth truncation, and perform them using TBLite.^37^

## 2 Theory

Here, we present an overview of GFN1-xTB for periodic systems. Atomic units are used throughout. The total GFN1-xTB energy is comprised of electronic (el), repulsive (rep), dispersion (disp) and halogen-bonding (XB) terms:
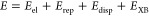
1The electronic energy is given by
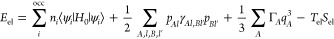
2where ψ_*i*_ are the valence molecular orbitals (MOs) with occupation numbers *n*_*i*_, and *H*_0_ is the zeroth-order Hamiltonian. A self-consistent treatment of the Coulomb repulsion resulting from charge density fluctuations is achieved through the incorporation of second and third-order terms.^11^ The second-order term is defined in terms of the shell-resolved partial charges, *p*_*Al*_, and a modified Coulomb interaction, γ_*Al*, *Bl*′_, where (*A*, *B*) and (*l*, *l*′) are indices for atomic centers and orbital shells, respectively. In molecular GFN1-xTB, γ is defined with the Klopman–Ohno (KO) functional form.^41^ The implications and limitations of this choice are the subject of the following section. The third-order term is included in a diagonal approximation, and defined in terms of partial atomic Mulliken charges, *q*_*A*_ = ∑_*l*_*p*_*Al*_, and the constant Γ_*A*_, which is the charge derivative of the atomic hardness parameter, η_*A*_.^42^ A detailed description of the remaining GFN1-xTB terms is given in the original paper.^9^

### 2.1 Periodic Systems

The GFN1-xTB method can be extended to periodic boundary conditions by expanding the atom-centered basis using a Bloch sum:^3^
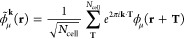
3In this expression, ϕ_μ_(**r** + **T**) defines an atom-centered orbital with composite state index μ = (*A*, *l*), located in a unit cell defined by the translation vector **T** = *n*_1_**a**_1_ + *n*_2_**a**_2_ + *n*_3_**a**_3_, where {**a**} define the real-space lattice vectors of the unit cell, and  are lattice multipliers. In the exponent, **k** defines the wave vector, and we use the convention that both forward and backward Fourier transforms contain a factor of 2 π. This convention is used throughout the manuscript.

Using the periodic basis set of Bloch functions, it is possible to construct periodic molecular orbitals:
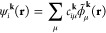
4and more generally, arbitrary operators of the form:
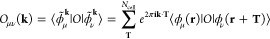
5where *c*_*i*μ_^**k**^ are the MO coefficients of state *i*. Using these quantities, one can obtain expectation values in a lattice by integrating over the reciprocal-space volume, *V*_*G*_, which in practice is approximated by a weighted discrete sum:^43^
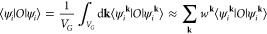
6

### 2.2 Zeroth-Order Hamiltonian and Overlap Matrices

Using eq 5, the zeroth-order Hamiltonian, overlap, and density matrices can be written, respectively, as
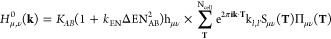
7
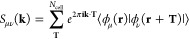
8
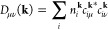
9where the Hückel constant matrix is *k*_*l*,*l*′_ = 1/2 (*k*_*l*_ + *k*_*l*′_) for onsite terms, and one otherwise, *h*_μν_ = 1/2 (*h*_*Al*_ + *h*_*Bl*′_) is the effective atomic energy level matrix, which implicitly includes a lattice sum as *h*_*Al*_ is defined in terms of the coordination number,^44^ Δ*EN*_*AB*_ = *EN*_*A*_ – *EN*_*B*_ defines the electronegativity matrix, and *n*_*i*_^**k**^ is the electronic occupation of MO state (*i*, **k**) . The definition of the Π matrix is given in Supporting Information (SI). 1. All parameters can be found in the official GFN-xTB Git repository,^45^ which amends several mistakes in the original publication.^9^

### 2.3 Second-Order Charge Fluctuations

The second-order Coulomb interaction in GFN1-xTB is defined using a modified version of the Klopman–Ohno (KO) function,^41^ which interpolates between the limits *R* → 0 and *R* → ∞:
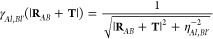
10The atomic hardness, η_*Al*, *Bl*′_, includes an orbital dependence, thereby introducing a dependence on the charge state of the atom into the second-order term.^46^ This is in addition to the modification of the electron–electron interaction on the atom in response to the charge state, which is captured by the diagonal of the third-order term.^42^

The use of the KO γ-function in periodic systems poses a challenge due to the lack of a closed-form solution for its Fourier transform. Additionally, its prior use in the electrostatics of DFTB+ resulted in convergence problems with the Ewald summation,^42,47^ and the incorporation of orbital-dependent atomic hardness parameters further adds complexity to the solution. The remainder of this section will be devoted to outlining a means of effectively partitioning eq 10 into short- and long-range components, such that we can solve the electrostatic contribution to eq 2 using standard lattice sum methods.

### 2.4 Binomial Expansion of the Klopman–Ohno γ-Function

A long-range interaction is one that is defined as falling off no faster than *R*^–*d*^, where *d* is the dimensionality of the system.^48^ In 3-dimensional periodic systems, a lattice sum over a uniformly distributed set of points, with an exponent *d* ≤ 3, is conditionally convergent or divergent. Truncating the potential at some arbitrary cutoff to allow its evaluation is therefore problematic. One of the main methods for dealing with this problem is to instead use a convergence function to partition the lattice sum in terms of two rapidly convergent sums, treated in real-space and reciprocal-space, respectively.^49^

The presence of the hardness parameter in eq 10 prevents us from Fourier transforming. Fortunately, we can expose the leading terms by expanding the KO γ-function using the generalized binomial theorem:
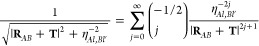
11Truncating eq 11 to terms where *j* ≤ 1 defines a long-range (LR) potential, which can also be Fourier-transformed. The short-range (SR) potential is then, by definition, the difference between the full potential (10) and the expansion of eq 11 up to *N*_*LR*_ terms:
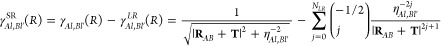
12In principle, *N*_*LR*_ = 1 is sufficient as it captures the *R* and *R*^3^ terms that constitute long-range interactions. This is shown for bulk MgO in SI. 4.1. We note that at this point, the formulation remains exact, and the number of terms in the binomial expansion simply act as a means of controlling the partitioning of the KO γ-function. Additionally, this scheme is general and can be applied to any damped potential function of the form |*R*^*n*^ + *c*|^–*m*^.^41,50,51^

### 2.5 Generalized Lattice Sum Expression for Arbitrary Powers of R

The total electrostatic energy associated with the KO γ-potential, eq 10, can be written as the sum of the SR and LR energy contributions, *E*_2nd_ = *E*_2nd_^SR^ + *E*_2nd_^LR^. Using eqs 2 and 12, the SR energy is defined as
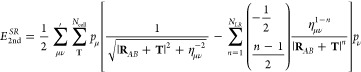
13having made the substitution *n* = 2*j* + 1 in eq 12, such that *n* defines the order of the potential. The prime in the summation denotes not to sum over *A* = *B* for **T** = 0, and the shell-resolved partial charge is defined as
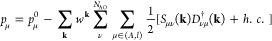
14where *w*^**k**^ are the weights associated with the **k**-grid, and *p*_*Al*_^0^ are reference shell charges defined according to molecular GFN1-xTB.^9^ By construction, eq 13 can be evaluated straightforwardly, using a real-space summation over translation vectors. The binomial expansion allows us to define a LR energy expression that can be evaluated using a generalized Ewald summation for arbitrary powers in *R*:
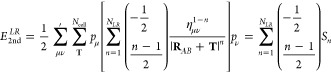
15where *S*_*n*_ is the electrostatic energy for a potential *R*^–*n*^. The definition for the electrostatic energy is analogous to the expression originally derived by Williams,^52^ but extended to include the shell-resolved partial charges and atomic hardness:
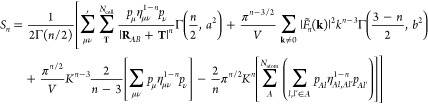
16where the convergence (or screening) function is defined by the incomplete gamma function, Γ(*m*, *x*), and normalized by the gamma function, Γ(*m*) . *K* defines the broadening parameter of the convergence function, and constants *a* and *b* are defined as *a*^2^ = π *K*^2^ |**R**_*AB*_ + **T**|^2^ and *b*^2^ = π *k*^2^/*K*^2^, respectively. The four terms in eq 16 correspond to energy contributions due to the real-space lattice sum of screened charges, the reciprocal-space sum of compensating charge density, the **k** = 0 term of the reciprocal-space sum, which is zero for *n* = 1 in a charge-neutral system, and the self-interaction correction to the compensating charge density. Finally, *F̃*_*n*_(**k**) defines the modified structure factor:
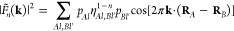
17Explicitly evaluating eq 16 for *R*^–1^, we obtain the standard textbook solution,^53^ as the exponent of the hardness parameter equals zero. This is given in SI. 3. The electrostatic energy for the *R*^–3^ term is similarly evaluated as
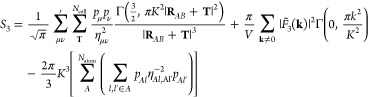
18where the third term of eq 16 is omitted, as it is undefined for *n* = 3. This poses a problem. As a consequence of the binomial expansion, the hardness parameter scales the shell charges for all terms with *n* > 1, leading to a nonzero net charge: ∑_μ ν_^*N*_atom_^*p*_μ_ η_μ_^1–*n*^, ν*p*_ν_ ≠ 0. This causes expression 18 to be divergent if the term is excluded. Nevertheless, it is also physically incorrect to completely ignore it, as doing so introduces a dependence of the potential on the choice of Ewald broadening, *K*. We provide a mathematical argument for the requirement of this term in SI. 4.2, and demonstrate the impact of neglecting it in Figure S2. The presence of a divergence therefore requires us to derive an approximation for the **k** = 0 term of the *R*^–3^ electrostatic energy contribution.

### 2.6 An Approximation to the *k* = 0 Contribution to the Electrostatic Energy

The real-space integral expression for the compensating charge density, as defined by Williams, is^52^
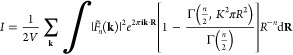
19Rather than Fourier transform eq 19, we define the real-space integral in the range [0, *r*_0_], where *r*_0_ is some constant that will go to infinity in the limit of the final solution. Evaluating eq 19 specifically for *n* = 3, at **k** = 0, we get:
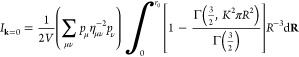
20This integral can be expressed by a generalized hypergeometric function (see SI. 4.3):
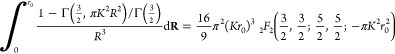
21which in the limit *R* → ∞ can be asymptotically expanded and approximated by the expansion’s leading term:
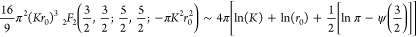
22where ln(*r*_0_) is the divergent term in the limit *r*_0_ → ∞, ψ(*x*) is the digamma function, and  approximately evaluates to 0.554. We also observe a logarithmic dependence on *K* in eq 22, which is consistent with what we would expect from integrating eq 20, in SI. 4.1

Equation 22 still requires some finite choice for *r*_0_ in order to be evaluated. A natural choice is to set *r*_0_ equal to one, such that the divergent term is set to zero and the result becomes material-independent. We note that *r*_0_ could alternatively be treated as an empirical fitting parameter to optimize the electrostatics, however, this approach would require fitting a unique parameter for each system studied. Substituting the convergent terms of eqs 22 into 20 finally gives
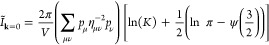
23which consequently allows us to fully evaluate eq 18 as
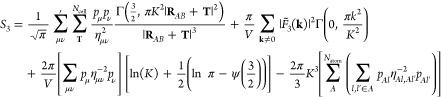
24Equation 24 retains the **k** = 0 contribution to the cubic term of the electrostatic energy, while maintaining independence of the potential with regards to the choice of broadening parameter, *K*. Equations 13, 15, 16 and 23 therefore rigorously define a modified, convergent KO γ-potential for GFN1-xTB.

## 3 Results and Discussion

In this section, we benchmark the performance of our periodic implementation of GFN1-xTB, referred to as QCore-xTB, on both molecular and bulk crystals, which exhibit a range of electrostatic interactions. QCore-xTB was developed as part of the entos QCore electronic structure package.^35^ A quantitative comparison between tight-binding and DFT is performed using the equation of state (EOS), which is straightforwardly obtained from total energy calculations. The EOS allows us to infer material properties such as the bulk modulus, and provides a simple means of comparison between xTB and DFT total energies for nonequilibrium lattice parameters, which has important implications for structural optimization and molecular dynamics. Finally, we demonstrate an application of QCore-xTB on a set of metal-oxide frameworks (MOFs).

For all systems, we compare QCore-xTB to both DFT at the level of GGA, and to periodic GFN1-xTB, where the real-space potential is smoothly forced to zero at some fixed cutoff. We refer to this as GFN1-xTB(s), and provide more details in SI. 2. DFT calculations were performed using Quantum Espresso (QE) version 7.1, the PBESOL^38^ functional, and pseudopotentials from the GBRV ultrasoft pseudopotential library.^54^ GFN1-xTB(s) calculations were performed with the library TBLite^37^ and the client code DFTB+ 22.1.^55^ TBLite includes both the GFN1-xTB and xTB2 models, however, we restrict ourselves to comparing to GFN1-xTB. GFN2-xTB extends the treatment of the second-order potential beyond the point charge approximation, using a multipole expansion.^56^ A multipole treatment of the potential results in a more physical representation of the charge density, enabling the model to capture charge deformation and polarization in a self-consistent manner. It is therefore expected to give better results for ionic systems, however, it would also greatly increase the complexity of the implementation required to treat the KO γ-potential in periodic systems. None of the calculations presented include spin-polarization^57^ or spin–orbit coupling,^58^ which were only recently introduced to the GFN-xTB framework. Their exclusion has no effect on our conclusions.

### 3.1 Equation of State Comparison Using the Delta Factor

A comparison of a material’s EOS, between two codes, is performed using the delta factor. This defines a quantitative metric of relative agreement between two EOS curves, and is defined as the RMS difference between two EOS over a ± 6% interval, centered on the equilibrium volume *V*_0_ (this requires the equilibrium volumes of the two calculations to be aligned):^59,60^
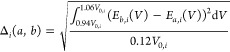
25The delta factor therefore gives an average relative difference in the total energy, about the equilibrium volume. For high-quality experimental data, one can expect a Δ ∼ 1 meV. This is comparable to a Δ ∼ 1–2 meV between DFT (GGA) calculations, however, it is important to recognize that this difference can increase by an order of magnitude depending on the choice and quality of pseudopotential, for example.^60^

### 3.2 Organic Molecular Crystals

For validation of QCore-xTB on molecular crystals, we employ the extensively studied X23 dataset.^61^ X23 is comprised of 23 experimentally well-defined, small, molecular crystals which are themselves comprised of rigid molecules. In general, we observe strong agreement between both tight-binding models and DFT, both in terms of equilibrium geometries and EOS curves. The equilibrium volumes and delta factors for both QCore-xTB and GFN1-xTB(s) are summarized in Table 1, and the corresponding EOS curves are shown in SI. Figure S3. The mean delta factor for QCore-xTB is 12 meV/atom. QCore-xTB also shows quantitative agreement with GFN1-xTB(s), implying that the Coulombic potential makes a relatively minor contribution to the total energy of organic crystals. This is what we would intuitively expect. Organic crystals retain their molecular character, exhibit shell charges with small magnitudes, and are held together by intermolecular interactions. The molecular nature of these crystals means that the current GFN1-xTB parametrization, and corresponding zeroth-order Hamiltonian, accurately describe the local chemical environment. In addition, the small magnitude of the shell charges limits the contribution to the total energy from the problematic second-order Coulomb term, as there is very little charge transfer. If we examine the relative contributions of each term to the xTB total energy, given by eq 1, we find that the zeroth-order term makes the most significant contribution, typically about 2 orders of magnitude larger than the electrostatics. Both periodic GFN1-xTB models are therefore well-suited for simulating molecular crystals composed of light elements.

**Table 1:** Equilibrium Volumes and Delta Factors Computed with QCore-xTB and GFN1-xTB(s), for the X23 Molecular Set^a^

	QCore-xTB	GFN1-xTB(s)	QCore-xTB	GFN1-xTB(s)
molecule	*V*_eq_ (Å^3^)	*V*_eq_ (Å^3^)	Δ (meV/atom)	Δ (meV/atom)
urea	141 (3.3)	140 (3.8)	9.8	10.1
uracil	440 (2.5)	439 (2.6)	9.2	9.8
trioxane	594 (1.7)	598 (1.1)	4.8	3.0
triazine	538 (3.0)	542 (2.4)	11.0	8.4
succinic	237 (2.3)	235 (3.0)	7.0	9.3
pyrazole	695 (2.7)	694 (2.8)	69.3	72.0
pyrazine	190 (2.5)	190 (2.4)	8.6	8.3
oxacb	154 (2.1)	153 (3.0)	7.5	10.9
oxaca	307 (1.9)	304 (2.7)	6.8	10.1
naph	335 (1.6)	335 (1.7)	5.4	5.8
imdazole	333 (2.6)	333 (2.6)	8.7	8.7
hexdio	268 (2.4)	268 (2.4)	6.8	6.8
hexamine	165 (1.6)	165 (1.5)	4.4	4.1
formamide	214 (3.2)	213 (3.7)	9.1	10.8
ethcar	233 (2.3)	233 (2.6)	6.2	7.0
cytosine	450 (2.4)	448 (2.7)	8.5	9.8
cyanamide	404 (4.1)	404 (4.2)	13.9	14.2
benzene	454 (1.9)	453 (2.1)	6.0	6.4
anthracene	444 (1.5)	443 (1.6)	5.2	5.6
ammonia	117 (6.4)	115 (7.9)	12.2	18.6
adaman	366 (1.2)	366 (1.3)	3.0	3.2
acetic	286 (2.5)	285 (2.9)	6.8	7.7
CO_2_	181 (6.8)	183 (5.4)	39.3	29.7
mean	(2.7)	(2.9)	11.7	12.2

a Percentage errors in the equilibrium volumes are given in parentheses, relative to the DFT equilibrium volumes. In all calculations, a k-sampling of (4, 4, 4) is used. Only small differences are observed between QCore-xTB and GFN1-xTB(s), with both methods giving a mean delta factor of 12 meV/atom for the X23 molecular dataset. Bulk moduli obtained from the fitting are given in Table S2 of the SI.

### 3.3 Bulk Binary Crystals

For bulk solids, we benchmark periodic QCore-xTB on a selection of prototypical elemental and binary semiconductors, with a range of band gaps. These include semiconductors from group IV (Si, Ge, C, graphite), group III–V (GaAs, GaN, c-BN, h-BN), group II–VI (CdSe, PbS), oxides (TiO_2_-rutile, WO_3_ ZnO, ZrO_2_), TMDCs (MoS_2_, WS_2_) and ionic crystals (NaCl, MgO). Si, Ge and diamond lattice parameters are taken from Yin and Cohen.^62^ All other entries were obtained from the Materials Project database.^63^ The structure IDs, calculation settings and automated workflow details are provided in the SI. 5.

The delta factors between the tight-binding and DFT calculations reflect the cumulative errors that arise from the approximations to the DFT total energy in the tight-binding formalism, however this comparison cannot identify the error associated with a given approximation. By also comparing the delta factors between GFN1-xTB(s) and QCore-xTB, we are able to isolate the effect of the treatment of the second-order potential on the equation of state. The equilibrium volumes and delta factors are summarized in Table 2.

**Table 2:** Equilibrium Volumes and Delta Factors of Bulk Systems, for QCore-xTB and GFN1-xTB(s), versus DFT^a^

	QCore-xTB	GFN1-xTB(s)	QCore-xTB	GFN1-xTB(s)
system	*V*_eq_ (Å^3^)	*V*_eq_ (Å^3^)	Δ (meV/atom)	Δ (meV/atom)
Ge	48.9 (7.0)	45.8 (0.2)	39.8	1.8
graphite	45.2 (0.3)	45.1 (0.5)	2.6	3.2
c-BN	11.8 (0.5)	11.5 (2.3)	2.4	11.2
diamond	11.1 (0.8)	11.1 (1.1)	5.6	7.1
h-BN	43.3 (0.0)	42.2 (2.5)	0.3	12.7
CdSe	118.7 (5.0)	118.7 (5.0)	15.4	18.4
WS_2_	122.9 (1.2)	125.1 (3.0)	7.4	19.7
TiO_2_ (rutile)	62.0 (0.1)	57.1 (7.8)	7.6	29.3
MoS_2_	133.0 (5.2)	136.7 (8.1)	25.2	46.0
PbS	46.1 (10.0)	49.3 (3.9)	106.7	30.3
Si	44.1 (10.0)	43.4 (8.2)	76.6	57.7
GaAs	48.1 (5.7)	47.5 (4.5)	23.5	17.0
ZrO_2_	37.6 (15.7)	28.0 (13.8)	81.7	259.6
NaCl	37.4 (15.3)	92.0 (108.3)	36.7	966.1
GaN	25.8 (13.2)	28.6 (25.4)	77.7	286.1
MgO	22.5 (20.1)	19.5 (3.9)	108.5	40.0
ZnO	63.2 (33.5)	55.8 (17.9)	230.0	218.5
WO_3_	215.6 (0.4)	N/A	6.7	N/A
mean	(8.0)	(12.7)	47.5	119.1

a Percentage errors in the equilibrium volumes are given in parentheses. For the systems studied, we find that QCore-xTB predicts a mean delta factor 47 (median 24) and GFN1-xTB(s) predicts a mean delta factor 119 (median 29). Bulk moduli obtained from the fitting are given in Table S2 of the SI.

In general, we see that covalent semiconductors with small charge transfer exhibit the best results. Conversely, we observe that crystals with strong ionic character, such as metal oxides, perform poorly. GFN1-xTB(s) gives a large range of delta factors for the systems studied, varying from 2 meV/atom to 966 meV/atom, with several values exceeding 200 meV/atom. The spread in delta factor values found using QCore-xTB is, however, considerably reduced. In particular, we observe that the extremal delta factors found with GFN1-xTB(s) are significantly improved for all but ZnO, MgO and PbS when using our treatment of the KO γ-potential.

**Figure 1:**
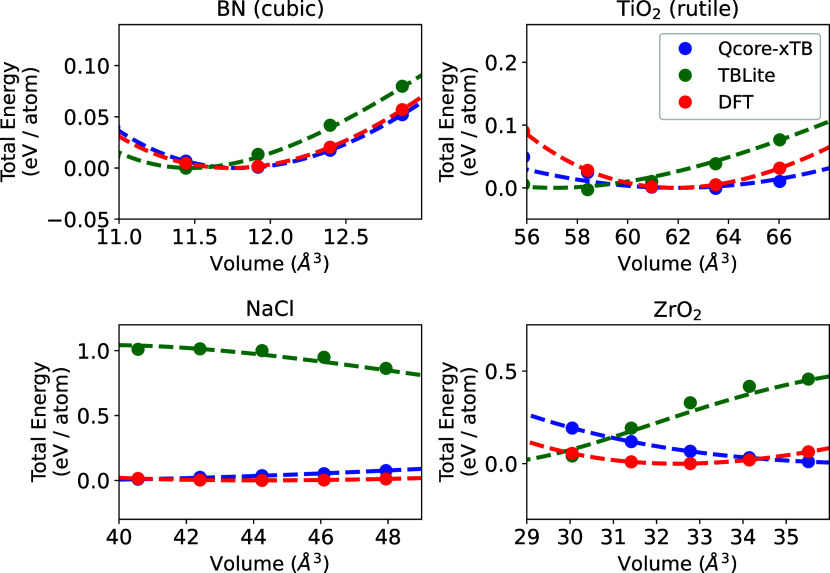
EOS data for c-BN, TiO_2_-rutile, NaCl and ZrO_2_ are presented using QCore-xTB (blue), GFN1-xTB(s) (green), and DFT (red). Data are displayed with points, while the model lines were fit using eq (22) in SI. 6. The *x*-axes of all plots range from 0.9 *V_eq_* to 1.1 *V*_eq_, where *V*_eq_ is the DFT equilibrium volume. Each *y*-axis is zeroed at the DFT total energy of the system at *V*_eq_.

Figure 1 shows the EOS curves for a selection of the studied bulk systems, demonstrating the range of agreement observed. The complete EOS data can be found in the SI Figure S4. c-BN exhibits excellent agreement, demonstrating DFT-level accuracy for both tight-binding models. For TiO_2_ rutile, we observe excellent improvement in the EOS about the equilibrium volume when using QCore-xTB. The two bottom panels, displaying EOS for NaCl and ZrO_2_, demonstrate the failure of the GFN1-xTB KO γ-potential for strongly ionic systems. Indeed, the curvature of the EOS for NaCl predicted by GFN1-xTB(s) is not physical. The use of QCore-xTB’s potential greatly improves agreement for both systems, particularly around the equilibrium volumes, indicating that a proper treatment of the electrostatics is essential for bulk solids.

From Table 2, it can be seen that QCore-xTB gives comparable or improved performance for all but MgO, Ge, Si and PbS. In the case of MgO, the delta factor of GFN1-xTB(s) is fortuitous. When we look at the EOS beyond ±6% about the equilibrum volume, shown in Figure S4 in the SI., we see that the behavior of MgO is unphysical. Similar unphysical behavior is observed for NaCl, ZnO, ZrO_2_ and WO_3_, all of which are also shown in Figure S4 in the SI.

The increased delta factors of Si and Ge given by QCore-xTB are harder to explain. There is no interatomic charge transfer in elemental semiconductors, leading one to expect electrostatics to play a minor role; however, the second-order electrostatic energy is still finite. One can also expect some intra-atomic redistribution of the charges due to their shell dependence, and the shell dependence of the hardness parameter. The closer agreement observed between GFN1-xTB(s) and the DFT reference for Si and Ge could arise from a cancellation of errors between the molecular parametrization and the treatment of the KO potential. Moreover, the Coulombic behavior, depending on the choice of softening, is only recovered at very large interatomic distances for KO potentials. This can result in ill-conditioned total energies with respect to the hardness parameters, where small changes in the parameters or the functional form of the potential may lead to variations in the total energy.^47^ Distinguishing whether discrepancies with DFT stem from errors associated with the use of the molecular parametrization for a specific bulk system or from the treatment of the KO potential is challenging. Consequently, making general assertions about which elemental combinations are likely to reproduce DFT results accurately, without performing preliminary benchmarks, remains a limitation of the current model.

A careful treatment of the KO γ-potential improves the qualitative behavior of the EOS curves and generally improves the equilibrium volumes of the bulk solids studied. However, it does not typically lead to quantitative agreement with DFT across a range of cell volumes. This outcome is expected for two reasons. First, an element-wise parametrization prioritizes transferability over accuracy, providing a reasonable description of a diverse range of elemental combinations and polymorphs at the expense of overall accuracy for any given system. This is observed in other element-wise parametrizations, such as the DFTB PTBP model of Cui et al.^14^ Second, GFN1-xTB was parametrized with a focus on metal–organic molecules. When the local chemical environments of the reference systems are not sufficiently similar to bulk solids, the parametrization will fail to reproduce the electronic structure. The transferability of the parametrization is likely the most important factor in improving the performance of GFN1-xTB for bulk systems.

### 3.4 Comparison with a Universal Force Field

The element-wise, additive formulation of GFN-xTB, with parametrizations for a large amount of the periodic table, constitutes a universal TB parametrization. It is therefore also pertinent to compare the relative error in QCore-xTB to the error associated with a machine-learnt universal force field (UFF). Unlike the delta factor, which gives an average difference between DFT and TB for relative changes in total energy over a fixed volume interval, the error in the total energy of a force field is defined as the difference in DFT total energy for a structure relaxed using DFT, and the same structure relaxed using the FF. Here, one obtains a single error in the total energy for the equilibrium structure (*V*_0_), found using the FF.

**Figure 2:**
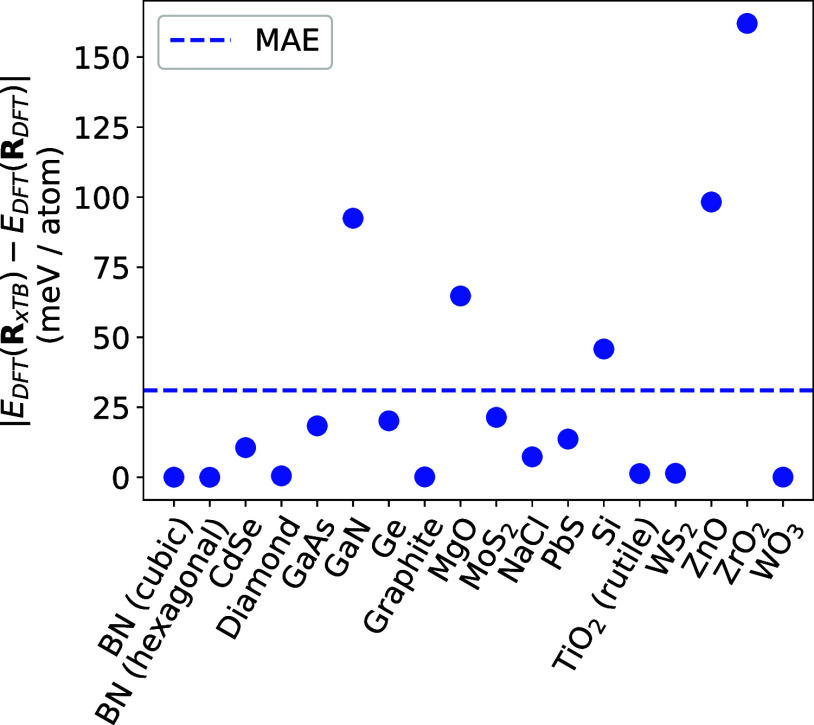
Absolute differences in DFT total energies for the bulk crystal dataset, computed using the DFT minimum-energy structure and the QCore-xTB minimum-energy structure, respectively.

We can perform this procedure by determining the equilibrium volume of a system computed with QCore-xTB from the EOS, given in Table 2, and running a single-point DFT calculation with the corresponding lattice parameters. The results are shown in Figure 2. Using QCore-xTB, we obtain a mean absolute error (MAE) for the bulk solid dataset of 31 meV/atom, which reduces to 16 meV/atom if we ignore the oxide outliers. In comparison, the machine-learnt UFF, M3GNet,^64^ was trained on a dataset containing elements 1–89, and was subsequently applied to 3140 structures from the Materials Project. This resulted in a total energy MAE of 35 meV/atom, with 80% of the systems exhibiting errors less than 28 meV/atom. Although our dataset is 2 orders of magnitude smaller, we observe a comparable MAE. This preliminary analysis demonstrates the potential of the GFN-xTB model as a universal TB parametrization for periodic systems, which may be beneficial in efficiently and accurately optimizing crystal structures. Investigation into the performance of structural relaxation with GFN-xTB is beyond the scope of this work, however we do provide the formal derivation of gradients and forces for periodic GFN1-xTB in SI. 7.

In principle, the simulation of ionic systems is not a limitation of third-order DFTB if the underlying parametrization has been fit on polymorphs with different bond lengths and coordination numbers.^65−67^ However, we are not able to say to what extent the disagreement between both xTB models and DFT can be attributed to the limited transferability of the current molecular GFN parametrization, versus the treatment of the second-order electrostatics. When considering the parametrization reference systems, the poor performance observed for Si and Ge, and the significant differences in delta factors between GaAs and GaN, it leads us to believe that the GFN1-xTB parametrization likely lacks transferability to bulk systems, at least for a subset of elements, or specific elemental combinations.

### 3.5 Application to Metal–Organic Frameworks

Finally, we demonstrate an application of QCore-xTB on metal–organic frameworks (MOFs). A MOF is a crystalline material that consists of metal ions or clusters, coordinated with organic ligands, resulting in the formation of a porous network. MOFs are characterized by their ultrahigh porosity, enormous internal surface areas, and high structural tunability.^68^ To date, more than 100,000 MOFs have been synthesized with a broad range of structural and chemical properties.^69^ This has led to a wide range of applications including gas separation and storage, sensing, and catalysis.^70^

**Figure 3:**
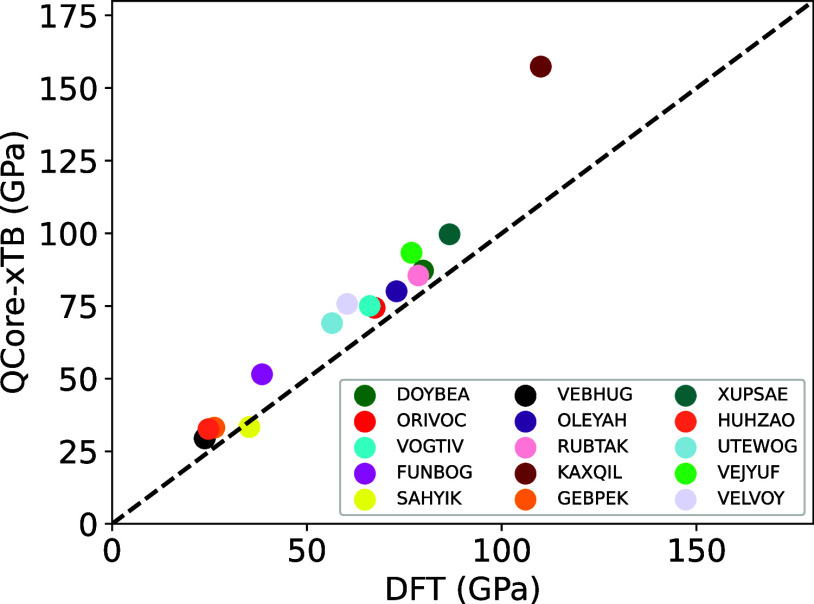
Comparison of bulk moduli for 15 common MOF structures, computed using QCore-xTB and DFT.

MOFs pose significant computational challenges for DFT calculations due to their large unit cell volumes, and the number of atoms within a typical unit cell, respectively. Semiempirical methods such as GFN-xTB offer a computationally less-demanding total energy method that may be more well-suited for performing structural relaxation, dynamics and screening studies of many systems. We select a random set of 15 closed-shell MOFs, compute their compression curves using both QCore-xTB and DFT, and compare their respective bulk moduli in Figure 3. Bulk moduli were obtained from fits using the Murnaghan equation of state, given by eq (22) in SI. 6. The application of QCore-xTB on this set of MOFs demonstrates consistent agreement with DFT and yields bulk moduli with a mean error of 12 GaP. As we do not relax the atomic positions after compression, we expect to slightly overestimate the bulk moduli with QCore-xTB. This may explain why all bulk moduli sit on or above the diagonal. The quality of the predictions for equilibrium geometries suggests that GFN1-xTB may be widely applicable to the simulation of MOF structural properties, without the need for reparameterisation, highlighting the potential for high-throughput optimization and design of novel frameworks using this method.

## 4 Conclusions

We have developed a binomial expansion of the second-order KO γ-potential as a means of partitioning short- and long-range interactions, in a form that enables the use of a generalized Ewald summation for the solution of the electrostatic energy in GFN1-xTB. This approach eliminates the need to smoothly force the Ewald lattice sum to zero at an arbitrary cutoff, and corrects unphysical behavior observed with existing implementations. Furthermore, the scheme is general and can be applied to any damped potential of the form, |*R*^*n*^ + *c*|^–*m*^.

Using QCore-xTB, we benchmarked our method against the X23 molecular crystal set and a selection of bulk semiconductors, comparing results with DFT (PBESOL) and GFN1-xTB(s). For molecular crystals, the electrostatic contribution to the total energy is minor, with both tight-binding models yielding a mean delta factor of 12 meV/atom. In bulk solids, this increases to 47 meV/atom, although QCore-xTB generally outperformed GFN1-xTB(s). Instances where our implementation performed less favorably are tentatively attributed to a cancellation of errors in GFN-xTB(s), and to the ill-conditioned nature of the electrostatic energy, which is inherent to this specific choice of γ-potential function.^47^ While these are the most likely sources of error, we refrain from overstating this conclusion due to the difficulty in disentangling the effects of the approximations on the KO potential from errors intrinsic to the parametrization. Despite this, the mean absolute error (MAE) in total energy found with QCore-xTB was 31 meV/atom, which is comparable to the MAE of the machine-learnt universal force field, M3GNet, making it suitable for performing structural relaxation.^64^

While our work generally enhances the accuracy and applicability of GFN1-xTB for periodic systems, our benchmarks also highlight the key limitations of using a shell-dependent KO γ-potential for the description of the electrostatics, and underscore the need for further improvements in future models. In particular, GFN-xTB would greatly benefit from a solids-specific parametrization, which would also facilitate the replacement of the functional form of the potential.

## Supporting Information Available

Materials Project IDs, calculation settings and EOS plots for all systems studied, as well as additional details on the asymptotic expansion of the hypergeometric function, and a full derivation of the gradient theory for periodic GFN1-xTB (PDF)
