# Supplementary material for: Periodic GFN1-xTB Tight Binding: A Generalized Ewald Partitioning Scheme for the Klopman–Ohno Function
Source: J Chem Theory Comput. 2025 Feb 5;21(4):1615–25. doi: 10.1021/acs.jctc.4c01234 (PMC11866747; doi:10.1021/acs.jctc.4c01234)
Supplement: Supplementary file 1 — ct4c01234_si_001.pdf [file ct4c01234_si_001.pdf]

Supporting Information: Periodic GFN1-xTB Tight-Binding: A Generalised Ewald Partitioning Scheme for the Klopman-Ohno Function

Alexander Buccheri,[*,†,‡,‖] Rui Li,[*,¶,‡] J. Emiliano Deustua,[¶,‡,⊥] S. Mohamad Moosavi,[§] Peter J. Bygrave,[†,#] and Frederick R. Manby[†,#]

†*School of Chemistry, University of Bristol, Cantocks Close, Bristol BS8 1TS, United Kingdom*

‡*These authors contributed equally to this work.*

¶*Division of Chemistry and Chemical Engineering, California Institute of Technology, Pasadena, CA 91125, United States of America*

§*Chemical Engineering and Applied Chemistry, University of Toronto, Toronto, Ontario M5S 3E5, Canada*

‖*Department of Phyics, Max Planck Institute for the Structure and Dynamics of Matter, Luruper Ch 149, 22761, Hamburg, Germany*

⊥*Current address: Examol Corporation, 5831 Forward Avenue, Suite 555, Pittsburgh, PA 15217, United States of America*

#*Current address: Iambic Therapeutics, 5627 Oberlin Drive, Suite 120, San Diego, CA 92121, United States of America*

E-mail: alexander.buccheri@mpsd.mpg.de; rli3@caltech.edu

Contents

List of Tables S-2

1 Zeroth-Order Hamiltonian Terms S-3

2 Smoothly Truncating the Real-Space Potential S-3

3 Lattice Sum Energy for the Coulomb Potential S-4

4 Klopman-Ohno Potential Partitioning S-5

4.1 Convergence of the Total Energy as a Function of Potential Order S-5

4.2 Dependence of the R^{-3} Ewald Summation on K S-6

4.3 Deriving an Expression for the Long Range Integral of the R^{-3} Term S-9

5 Calculation Settings and Workflow S-10

6 Equation of States S-11

7 Derivation of the Gradients in Periodic GFN1-xTB S-12

7.0.1 Pulay Force S-12

7.0.2 Gradient of the Hamiltonian S-18

Gradients for the Electrostatic Terms S-19

References S-21

List of Tables

S1 Materials Project IDs for bulk systems S-10

S2 Bulk moduli fits to X23 molecular crystal EOSs S-14

S3 Bulk moduli fits to bulk crystal EOSs S-16

1 Zeroth-Order Hamiltonian Terms

In periodic GFN1-xTB, the Π matrix is defined as:

$$\Pi(|\mathbf{R}_{AB}+\mathbf{T}|) = \left[1 + k_{A,l}^{\text{poly}}\left[\frac{|\mathbf{R}_{AB}+\mathbf{T}|}{R_{\text{cov},AB}}\right]^{1/2}\right]\left[1 + k_{B,l'}^{\text{poly}}\left[\frac{|\mathbf{R}_{AB}+\mathbf{T}|}{R_{\text{cov},AB}}\right]^{1/2}\right]. \tag{1}$$

where $k_{A,l}^{\text{poly}}$ and $k_{B,l'}^{\text{poly}}$ are element-specific parameters. The effective atomic energies, $h_{\mu,\nu}$, implicitly depend upon the translation vector through the modified D3 coordination number:[S1]

$$h_{\mu,\nu} = \frac{1}{2}(h_{Al} + h_{Bl'}), \tag{2}$$

where:

$$h_{Al} = H_{Al}\left(1 + k_{\text{CN},l}\text{CN}_{\text{A}}\right), \tag{3}$$

and the modified D3 coordination number is:

$$\text{CN}_A = \sum_{\mathbf{T}}\sum_{B\neq A}^{\text{CN}_{\text{cutoff}}}\left[1 + e^{-k_1\left(\frac{R_{cov,AB}}{|\mathbf{R}_{AB}+\mathbf{T}|}-1\right)}\right]^{-1}. \tag{4}$$

Here, $R_{\text{cov,AB}} = (r_{\text{cov,A}} + r_{\text{cov,B}})$ is the covalent distance, r_{cov} is the covalent atomic radius,[S1,S2] and $k_{\text{CN},l}$ are global scaling parameters for s, p and d shells. The coordination number cutoff, $\text{CN}_{\text{cutoff}}$, is defined to be 40 Bohr in accordance with the parameterization scheme.[S1,S2] We also note that the atomic separation vector is defined as $\mathbf{R}_{AB} = \mathbf{R}_A - \mathbf{R}_B$.

2 Smoothly Truncating the Real-Space Potential

A simple approach to treating long-range components of the KO gamma-potential is to smoothly scale the real-space lattice sum to zero at some arbitrary cutoff, r_c. Existing

periodic implementations[S3,S4] use a cubic Hermite polynomial of the form:

$$f_{\text{smooth}}(r/\delta r) = \begin{cases} 1, & \text{if } r < (r_c - \delta r), \\ 0, & \text{if } r > r_c, \\ -6\left(\frac{r_c}{\delta r}\right)^5 + 15\left(\frac{r_c}{\delta r}\right)^4 - 10\left(\frac{r_c}{\delta r}\right)^3 + 1, & \text{if } (r_c - \delta r) \leq r_1 \leq r_c, \end{cases} \tag{5}$$

to achieve this. The function and its first two derivatives are continuous at $r/\delta r = 0$ and $r/\delta r = 1$. Eq. (5) smoothly interpolates between 1 and 0 from $r = (r_c - \delta r)$ to $r = r_c$, allowing one to scale the potential over a length δr:

$$\gamma^{\text{SR}}_{Al,Bl'}(R) = f_{\text{smooth}}\left(\frac{r}{\delta r}\right)\left[\frac{1}{\sqrt{|\mathbf{R}_{AB} + \mathbf{T}|^2 + \eta^{-2}_{Al,Bl'}}} - \frac{1}{|\mathbf{R}_{AB} + \mathbf{T}|}\right]. \tag{6}$$

In doing so, one discards part of the contribution from the long-range component, which would otherwise be in:

$$\gamma_{Al,Bl'}(R) = \left[\frac{1}{\sqrt{|\mathbf{R}_{AB} + \mathbf{T}|^2 + \eta^{-2}_{Al,Bl'}}} - \frac{1}{|\mathbf{R}_{AB} + \mathbf{T}|}\right], \tag{7}$$

changing the electrostatic energy, and affecting the final result. δr is set to one Bohr in both TBLite and CP2K, and r_c is set to 10 Bohr in TBLite.

3 Lattice Sum Energy for the Coulomb Potential

The electrostatic energy can be expressed as the sum of two converging series:

$$S_1 = \frac{1}{2}\sum_{\mu\nu}{}' \sum_{\mathbf{T}}^{N_{\text{cell}}} p_\mu p_\nu \frac{\text{erfc}(\sqrt{\pi}K|\mathbf{R}_{AB} + \mathbf{T}|)}{|\mathbf{R}_{AB} + \mathbf{T}|} + \frac{1}{2\pi V}\sum_{\mathbf{k}\neq 0} \frac{|\tilde{F}_1(\mathbf{k})|^2}{k^2} e^{\frac{-\pi k^2}{K^2}} - K\sum_{A}^{N_{\text{atom}}}\left(\sum_l p_{Al}\right)^2, \tag{8}$$

where we have used the convention that both forward and backward FTs contain a factor of 2π in the exponent, such that we remain consistent with the convention of Williams.[S5] $\tilde{F}_1(\mathbf{k})$ defines the structure factor, given in the main text.

4 Klopman-Ohno Potential Partitioning

4.1 Convergence of the Total Energy as a Function of Potential Order

Theoretically, the electrostatic energy—and consequently the total energy—is expected to converge once the $n = 1$ and $n = 3$ terms are subtracted from the KO potential. This subtraction defines a short-range electrostatic energy expression:

$$E^{SR}_{\text{2nd}} = \frac{1}{2}\sum_{\mu\nu}{}' \sum_{\mathbf{T}}^{N_{\text{cell}}} p_\mu \left[\frac{1}{\sqrt{|\mathbf{R}_{AB}+\mathbf{T}|^2+\eta_{\mu\nu}^{-2}}} - \sum_{n=1}^{N_{LR}=2} \binom{-\frac{1}{2}}{\frac{n-1}{2}} \frac{\eta_{\mu\nu}^{1-n}}{|\mathbf{R}_{AB}+\mathbf{T}|^n} \right] p_\nu, \tag{9}$$

$$= \frac{1}{2}\sum_{\mu\nu}{}' \sum_{\mathbf{T}}^{N_{\text{cell}}} p_\mu \left[\frac{1}{\sqrt{|\mathbf{R}_{AB}+\mathbf{T}|^2+\eta_{\mu\nu}^{-2}}} - \frac{1}{|\mathbf{R}_{AB}+\mathbf{T}|} + \frac{\eta_{\mu\nu}^{-2}}{2|\mathbf{R}_{AB}+\mathbf{T}|^3} - \frac{6\eta_{\mu\nu}^{-4}}{8|\mathbf{R}_{AB}+\mathbf{T}|^5} \pm \dots \right] p_\nu, \tag{10}$$

which is evaluated only in real-space. The terms subtracted from Eq. (9) consequently define a long-range potential, with each term evaluated using an Ewald summation. Figure S1 illustrates this behaviour, showing the convergence of the total energy as a function of the real-space sum cutoff, $|\mathbf{T}| \leq |\mathbf{T}_{\text{max}}|$, in Eq. (9). The maximum order represents the maximum R^{-n} used to define the long-range potential (and therefore subtracted from the short-range potential).

In particular, the blue data highlights the divergence caused by the R^{-3} component of

the short-range potential when the maximum order is $n = 1$. For large R, the divergence exhibits a logarithmic trend. Once the $n = 3$ term is subtracted from Eq. (9), the total energy converges, indicating that $\gamma^{\mathrm{SR}}_{Al,Bl'}(R)$ no longer contains long-range contributions.

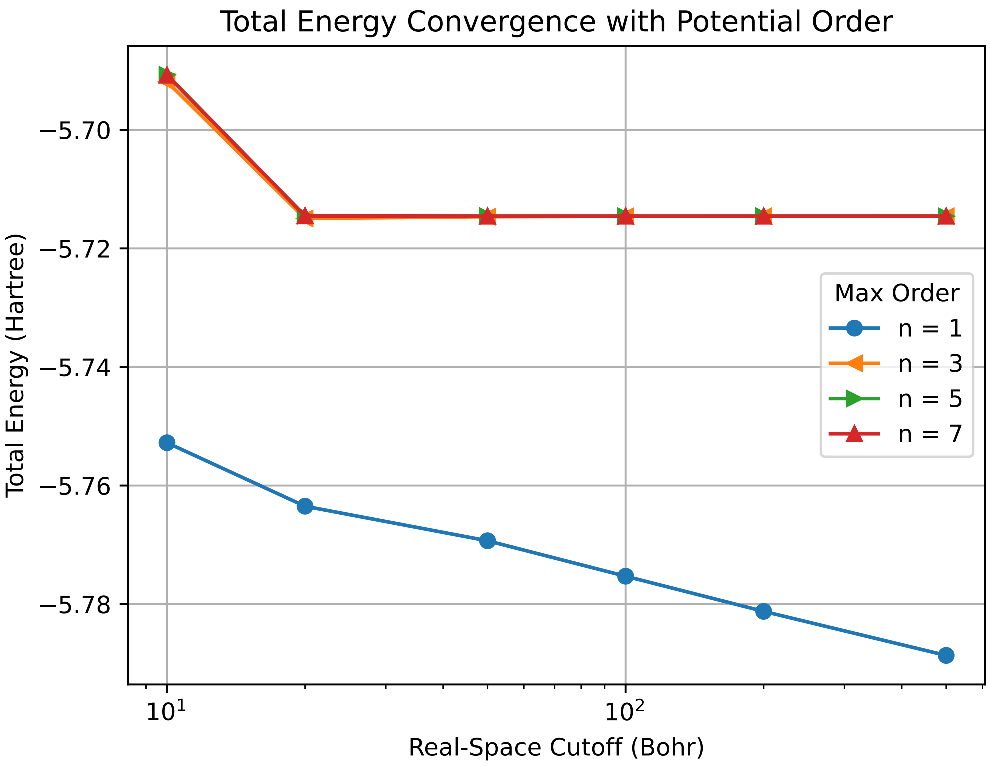

Figure S1: Convergence in the total energy of bulk MgO as a function of the real-space Ewald cutoff, for several maximum potential orders. The total energy of the system is consistent for $n \geq 3$.

4.2 Dependence of the R^{-3} Ewald Summation on K

To determine whether the R^{-3} Ewald sum is independent of the broadening parameter, K, we can take the derivative of the Ewald potential with respect to K and set it equal to zero:

$$\frac{\partial V_{\mathrm{Ewald}}(\mathbf{R})}{\partial K} = \frac{1}{\sqrt{\pi}} \sum_{\mathbf{T}\in\mathcal{L}} \frac{1}{|\mathbf{R}+\mathbf{T}|^3} \frac{\partial \Gamma\left(\frac{3}{2}, \pi K^2 |\mathbf{R}+\mathbf{T}|^2\right)}{\partial K} + \frac{\pi}{V} \sum_{\mathbf{k}\in\mathcal{L}^*} k^0 e^{2\pi i \mathbf{k}\cdot\mathbf{R}} \frac{\partial \Gamma\left(0, \frac{\pi k^2}{K^2}\right)}{\partial K} = 0, \tag{11}$$

where $\mathcal{L} = \{\mathbf{T} = n_1\mathbf{a}_1 + n_2\mathbf{a}_2 + n_3\mathbf{a}_3 \mid n_1, n_2, n_3 \in \mathbb{Z}\}$ defines the real-space lattice, $\mathcal{L}^*$ analogously defines the reciprocal-space lattice, and $\mathbb{Z}$ defines a set of integers over which both lattice sums run over. As such, the $\mathbf{k} = 0$ term is retained in the reciprocal-space sum, such that both summations include all terms. This proof focuses on balancing the contributions of the real-space and reciprocal-space summations for the R^{-3} potential, in the absence of any shell charges (and corresponding η). If the derivatives of the real-space and reciprocal-space summations balance, then the Ewald summation is independent of K.

Making use of the definition of the incomplete gamma function, its derivative is:[S6]

$$\frac{\partial\Gamma\left(s, x\right)}{\partial x} = -x^{s-1}e^{-x}. \tag{12}$$

Using Eq. (12) and the chain rule:

$$\frac{\partial\Gamma\left(\frac{3}{2}, \pi K^2|\mathbf{R}+\mathbf{T}|^2\right)}{\partial K} = -2\pi^{3/2}K^2|\mathbf{R}+\mathbf{T}|^3 e^{-\pi K^2|\mathbf{R}+\mathbf{T}|^2}, \tag{13}$$

such that the first term in Eq. (11) is:

$$\frac{1}{\sqrt{\pi}}\sum_{\mathbf{T}\in\mathcal{L}}\frac{1}{|\mathbf{R}+\mathbf{T}|^3}\frac{\partial\Gamma\left(\frac{3}{2}, \pi K^2|\mathbf{R}+\mathbf{T}|^2\right)}{\partial K} = -2\pi K^2\sum_{\mathbf{T}\in\mathcal{L}^*} e^{-\pi K^2|\mathbf{R}+\mathbf{T}|^2}. \tag{14}$$

Similarly:

$$\frac{\partial\Gamma\left(0, \frac{\pi k^2}{K^2}\right)}{\partial K} = \frac{2}{K}e^{-\frac{\pi k^2}{K^2}}, \tag{15}$$

such that the second term in Eq. (11) is:

$$\frac{\pi}{V}\sum_{\mathbf{k}\in\mathcal{L}} e^{2\pi i\mathbf{k}\cdot\mathbf{R}}\frac{\partial\Gamma\left(0, \frac{\pi k^2}{K^2}\right)}{\partial K} = \frac{2\pi}{VK}\sum_{\mathbf{k}\in\mathcal{L}^*} e^{-\frac{\pi k^2}{K^2}} e^{2\pi i\mathbf{k}\cdot\mathbf{R}}. \tag{16}$$

The derivative of the Ewald potential with respect to K can therefore be written as:

$$\frac{\partial V_{\text{Ewald}}(\mathbf{R})}{\partial K} = -K^2 \sum_{\mathbf{T}\in\mathcal{L}} e^{-\pi K^2|\mathbf{R}+\mathbf{T}|^2} + \frac{1}{VK}\sum_{\mathbf{k}\in\mathcal{L}^*} e^{-\frac{\pi k^2}{K^2}} e^{2\pi i\mathbf{k}\cdot\mathbf{R}} = 0, \tag{17}$$

such that:

$$K^2 \sum_{\mathbf{T}\in\mathcal{L}} e^{-\pi K^2|\mathbf{R}+\mathbf{T}|^2} = \frac{1}{VK}\sum_{\mathbf{k}\in\mathcal{L}^*} e^{-\frac{\pi k^2}{K^2}} e^{2\pi i\mathbf{k}\cdot\mathbf{R}}. \tag{18}$$

Rearranging, and substituting for $K = \sqrt{\frac{\alpha}{\pi}}$, one obtains:

$$\sum_{\mathbf{T}\in Z} e^{-\alpha|\mathbf{R}+\mathbf{T}|^2} = \frac{1}{V}\left(\frac{\pi}{\alpha}\right)^{3/2} \sum_{\mathbf{k}\in\mathcal{L}^*} e^{-\frac{\pi^2 k^2}{\alpha}} e^{2\pi i\mathbf{k}\cdot\mathbf{R}}, \tag{19}$$

which is the definition of the Poisson substitution formula for a Gaussian, $e^{-\alpha|\mathbf{x}|^2}$.[S7] This proves that the real- and reciprocal-space sums balance, meaning that the Ewald potential for $n = 3$ is independent of K when all terms are present in both summations. When the electrostatic energy is defined, one avoids the self-interaction in the real-space sum, and subtracts it from the reciprocal-space sum, such that the terms remain balanced. However, if the $\mathbf{k} = 0$ term is not present, there is not a complete cancellation and a dependence on K is introduced. The functional form of this dependence can be understood by explicitly evaluating Eq. (16) at $\mathbf{k} = 0$, for $n = 3$:

$$\frac{2\pi}{VK}\sum_{\mathbf{k}=0} e^{-\frac{\pi k^2}{K^2}} e^{2\pi i\mathbf{k}\cdot\mathbf{R}} = \frac{2\pi}{V}\frac{1}{K}. \tag{20}$$

In the instance of net zero shell charges, the $\mathbf{k} = 0$ term does not contribute, however in our formulation the shell charges are scaled by the hardness parameter due to the binomial expansion, leading to a finite net charge. Figure S2 shows the effect of not including the $\mathbf{k} = 0$ term on the total energy, as a function of the broadening parameter K, for a maximum binomial expansion of $n = 5$ ($N_{LR} = 3$). The dependence in the R^{-3} term is shown in orange. Regardless of the number of terms that are subtracted from $\gamma^{\text{SR}}_{Al,Bl'}(R)$, the total energy

remains dependent on K if the $\mathbf{k} = 0$ term is excluded from *any* order of the potential.

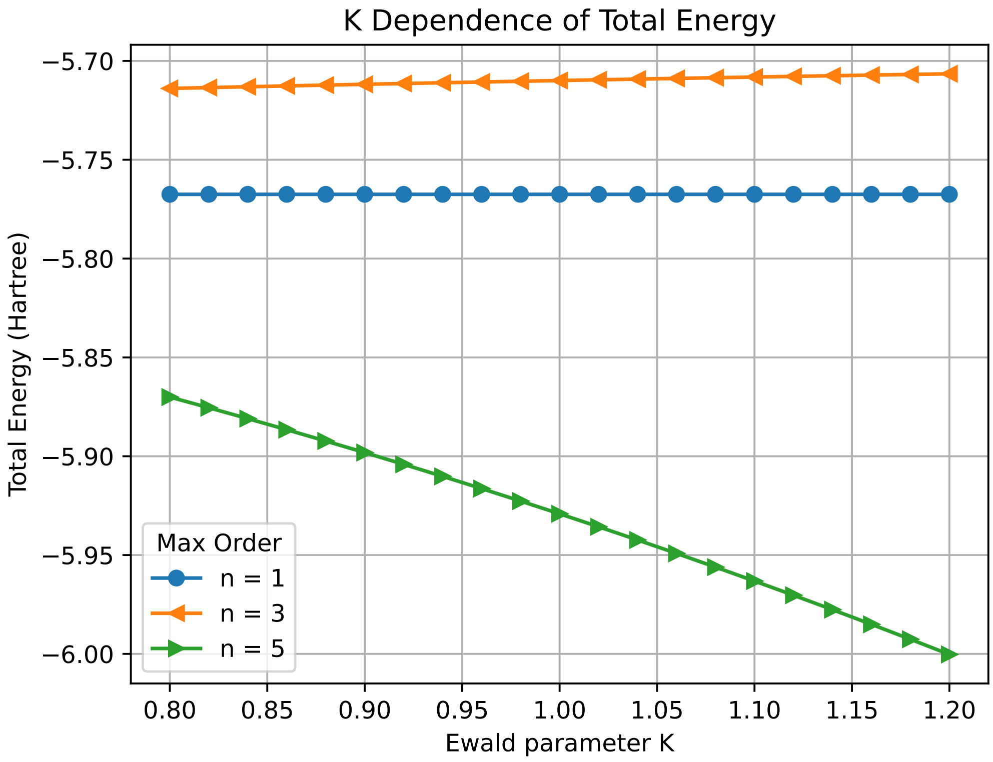

Figure S2: Dependence of the total energy on the Ewald broadening parameter, K, when the $\mathbf{k} = 0$ term is excluded from the Ewald summation. This dependence is present irrespective of the maximum order of the terms used to define $\gamma^{\text{SR}}_{Al,Bl'}(R)$.

4.3 Deriving an Expression for the Long Range Integral of the R^{-3} Term

Relating the integral:

$$\int_0^{r_0} \frac{1 - \Gamma(\frac{3}{2}, \pi K^2 r^2)/\Gamma(\frac{3}{2})}{r^3} d\mathbf{r} \tag{21}$$

to the hypergeometric function, $F_2(a, b, c, d; z)$, can be performed using Mathematica:[S8]

```
Integrate[4 Pi (1 - Gamma[3/2, Pi K^2 r^2 ]/ Gamma[3/2])/r, {r, 0, r0}]
```

and the asymptotic expansion in the limit $Kr_0 \to \infty$ is found with the following code:

```
Limit[16/9 Pi^2 kr0^3 HypergeometricPFQ[{3/2, 3/2}, {5/2, 5/2}, -Pi kr0^2]
```

```
− 4 Pi Log[kr0], kr0 −> Infinity]
```

5 Calculation Settings and Workflow

For most crystals, the lattice parameters and atomic positions are taken from entries in the Materials Project database.[S9] Their MP IDs are given in Table S1. Silicon, diamond and germanium lattice constants were taken from Yin and Cohen.[S10] Convergence studies for all

Table S1: Materials Project IDs for the bulk systems in this study.

**Crystal**	**MP-ID**
ZnO	2133
MoS_2	2815
WS_2	224
BN (hex)	2653
BN (cubic)	1639
MgO	1265
NaCl	22862
ZrO_2	1565
PbS	21276
TiO_2 (rutile)	2657
CdSe	1070
GaN	830
Graphite	48
GaAs	2534
WO_3	19033

systems and codes were performed automatically, using a python workflow based on top of Atomic Simulation Environment (ASE) classes.[S11] This provided a consistent means with which to handle k-grid convergence and results comparison. Our python workflow, along with the data analysis, can be found on Github.[S12] A regular Monkorst Pack grid was used for all calculations.[S13] Grid sampling was determined in a naive manner, using the relative magnitude of the reciprocal-space lattice vectors to determine the relative sampling per k-dimension. While better algorithms exist, we note that the choice of sampling scheme only impacts the efficiency of a calculation so long as the total energy is converged with respect

to it. For single-point calculations on small unit cells, performed with tight-binding or DFT (GGA), achieving optimal k-sampling is not a concern.

The total energy of all calculations was converged to within 1 meV with respect to the k-point sampling, and used an SCF (SCC) tolerance of 1×10^{-6}Ha (0.16 meV). Furthermore, the converged k-sampling from each GFN1-xTB(s) calculation is used as a starting point for the corresponding DFT calculation. In this way, we ensure that our final results are independent of the specific choice of k-grid, and we simply compare calculations converged to a consistent precision. QCore-xTB and GFN1-xTB(s) calculations were run with an electron temperature of 300 K, whereas DFT was run at 0 K, however the entropy term, $-T_{el}S_{el}$, contributes $\leq$ 0.1meV to the total energy for all systems studied, and so does not affect the results.

To reduce the total number of DFT calculations we chose to fix the plane wave cutoff at an arbitrarily high value of 120 Ry, and validate *a posteriori*. This is equivalent to 1633 eV, which is well in excess of the 1000 eV cutoff used by Reilly and Tkatchenko.[S14] They found a 1000 eV cutoff to be sufficient to converge both norm-conserving and ultra-soft pseudopotential total energy calculations to less than 1 meV per atom, for the X23 molecular dataset. For all systems of interest, we find that 120 Ry is sufficient to converge the total energy.

6 Equation of States

In this section, we present all EOS plots for the molecular crystals and bulk solids studied. Plots are shown over an interval $[0.9V_0, 1.1V_0]$, a volume range that is considerably larger than interval over which the delta factors are computed, to provide greater context. EOS are fit with the Birch-Murnaghan expression:[S15]

$$E(V) = E_0 + \frac{9V_0B_0}{16}\left\{\left[\left(\frac{V_0}{V}\right)^{2/3} - 1\right]^3 B_1 + \left[\left(\frac{V_0}{V}\right)^{2/3} - 1\right]^2 \left[6 - 4\left(\frac{V_0}{V}\right)^{2/3}\right]\right\} \quad (22)$$

where V is the volume of the crystal, V_0 is the volume at which the total energy $E(V)$ is minimised, B_0 is the bulk modulus, and B_1 is the first pressure derivative of the bulk modulus. The bulk moduli extracted from the EOS fits are reported in Tables S2 and S3 for the X23 and bulk crystal datasets, respectively.

Fig. S3 shows EOS curves for all molecular crystals in the X23 dataset, comparing QCore-xTB (blue) to DFT (red). Results for GFN1-xTB(s) are not shown as they are visually indistinguishable to QCore-xTB for most of the crystals. All calculations are performed with a k-sampling of $(4, 4, 4)$. Whilst gamma-point sampling was sufficient to obtain an error of $\leq$ 2meV per atom in the delta-factor for most molecular crystals, the higher k-sampling was required for ammonia, acetic, oxacb, cytosine, pyrazine, formamide and uracil. uracil exhibited the largest error due to gamma-point sampling, of 11.31 meV per atom.

For all bulk systems computed with QCore-xTB, with the exception of NaCl and TiO2, a real-space cut-off of 40 Bohr was used for summations over translation vectors, $\sum_{\mathbf{T}}^{N_{\text{cell}}}$. NaCl and TiO2 used a cut-off of 20 Bohr. We found all calculations to be converged to $\leq$ 1meV for a cut-off of 40 Bohr. TBLite's default real-space cut-off was used.

7 Derivation of the Gradients in Periodic GFN1-xTB

7.0.1 Pulay Force

The gradient of the energy is expressed as:

$$\frac{\partial E}{\partial \mathbf{X}} = \sum_{\mathbf{k}} w(\mathbf{k}) \left[\text{tr} \left(\left. \frac{\partial \mathbf{F}(\mathbf{k})}{\partial \mathbf{X}} \right|_{\mathbf{D}} \mathbf{D}(\mathbf{k}) \right) + \text{tr} \left(\mathbf{F}(\mathbf{k}) \left. \frac{\partial \mathbf{D}(\mathbf{k})}{\partial \mathbf{X}} \right|_{\mathbf{F}} \right) \right], \tag{23}$$

where $\mathbf{X}$ can be elements of the atom coordinates ($\mathbf{R}_C$) or the lattice vectors ($\mathbf{L}$) that determines the whole system. The contribution from the gradient of density matrix is the Pulay Force. We start by recalling the SCF equation at each k-point:

$$\mathbf{F}(\mathbf{k})\mathbf{C}(\mathbf{k}) = \mathbf{S}(\mathbf{k})\mathbf{C}(\mathbf{k})\varepsilon(\mathbf{k}). \tag{24}$$

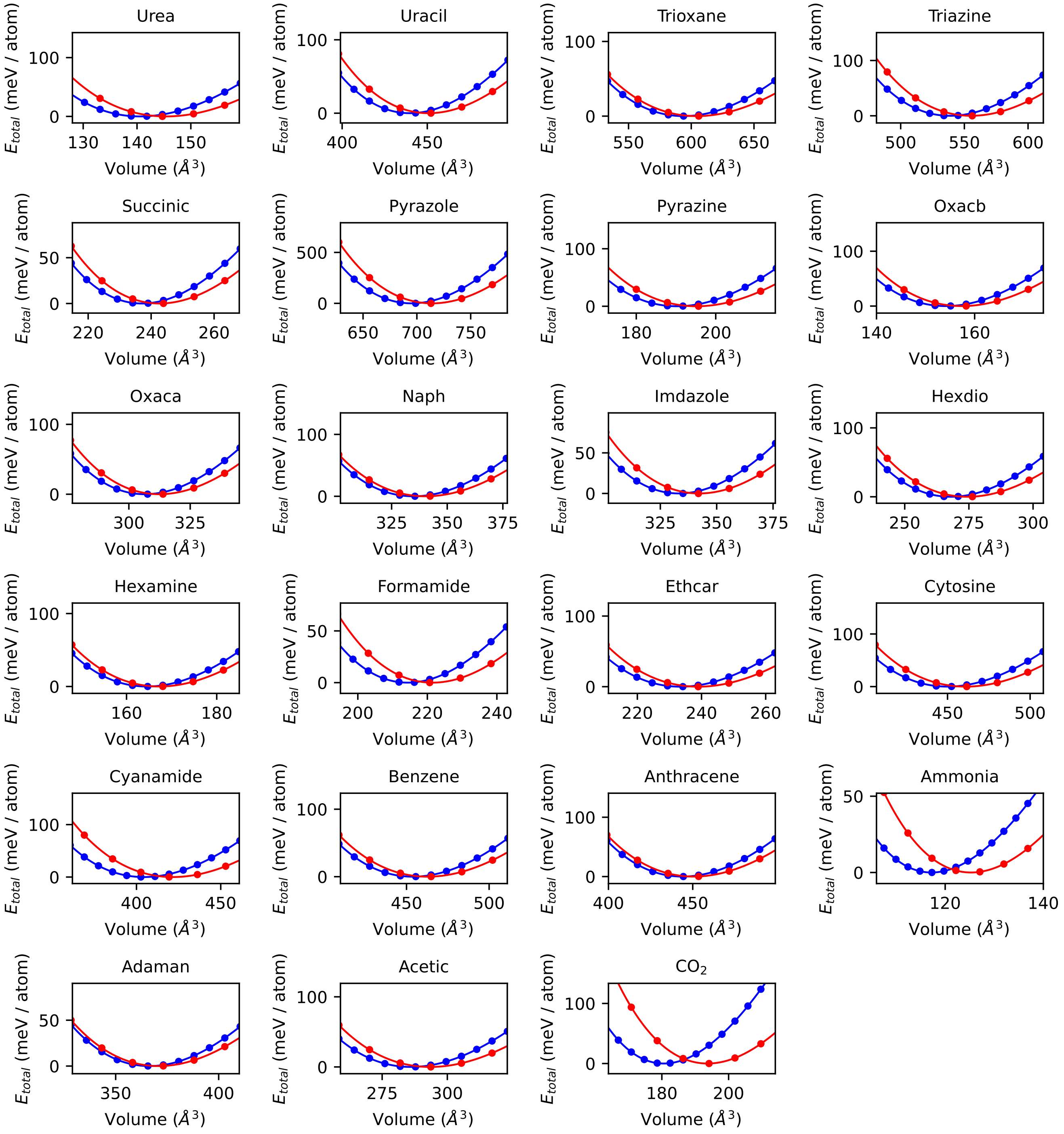

Figure S3: Equation of state curves for the X23 molecular benchmark set, comparing QCore-xTB (blue) to DFT (red). In all plots, the x-axis is centered on the DFT equilibrium volume, ranging from $0.9V_0$ to $1.1V_0$, and the y-axis is zeroed at the DFT total energy of the system at V_0.

Table S2: Bulk moduli (in GaP) of the X23 molecular crystal dataset, computed with QCore-xTB, GFN1-xTB(s) and DFT. The relative errors (%) of the tight-binding values w.r.t. DFT are defined as $100|B_{0,TB} - B_{0,DFT}|/B_{0,DFT}$, and are given in parentheses.

System	QCore-xTB	GFN1-xTB(s)	DFT
urea	143 (7)	128 (4)	133
uracil	180 (9)	182 (11)	165
trioxane	148 (16)	148 (17)	127
triazine	158 (10)	153 (6)	144
succinic	159 (11)	159 (11)	143
pyrazole	147 (9)	147 (9)	135
pyrazine	158 (11)	157 (11)	142
oxacb	168 (9)	167 (9)	153
oxaca	169 (9)	170 (10)	155
naph	164 (13)	165 (14)	145
imdazole	156 (8)	156 (9)	143
hexdio	152 (13)	151 (13)	134
hexamine	161 (9)	162 (9)	148
formamide	134 (8)	136 (10)	124
ethcar	130 (10)	129 (10)	118
cytosine	181 (8)	182 (9)	167
cyanamide	137 (8)	136 (6)	127
benzene	144 (13)	145 (14)	127
anthracene	174 (14)	174 (14)	153
ammonia	90 (5)	107 (25)	86
adaman	162 (13)	162 (13)	143
acetic	132 (10)	129 (7)	120
CO_2	129 (18)	127 (16)	109
Mean Relative Error (%)	10	11	
Median Relative Error (%)	10	10	

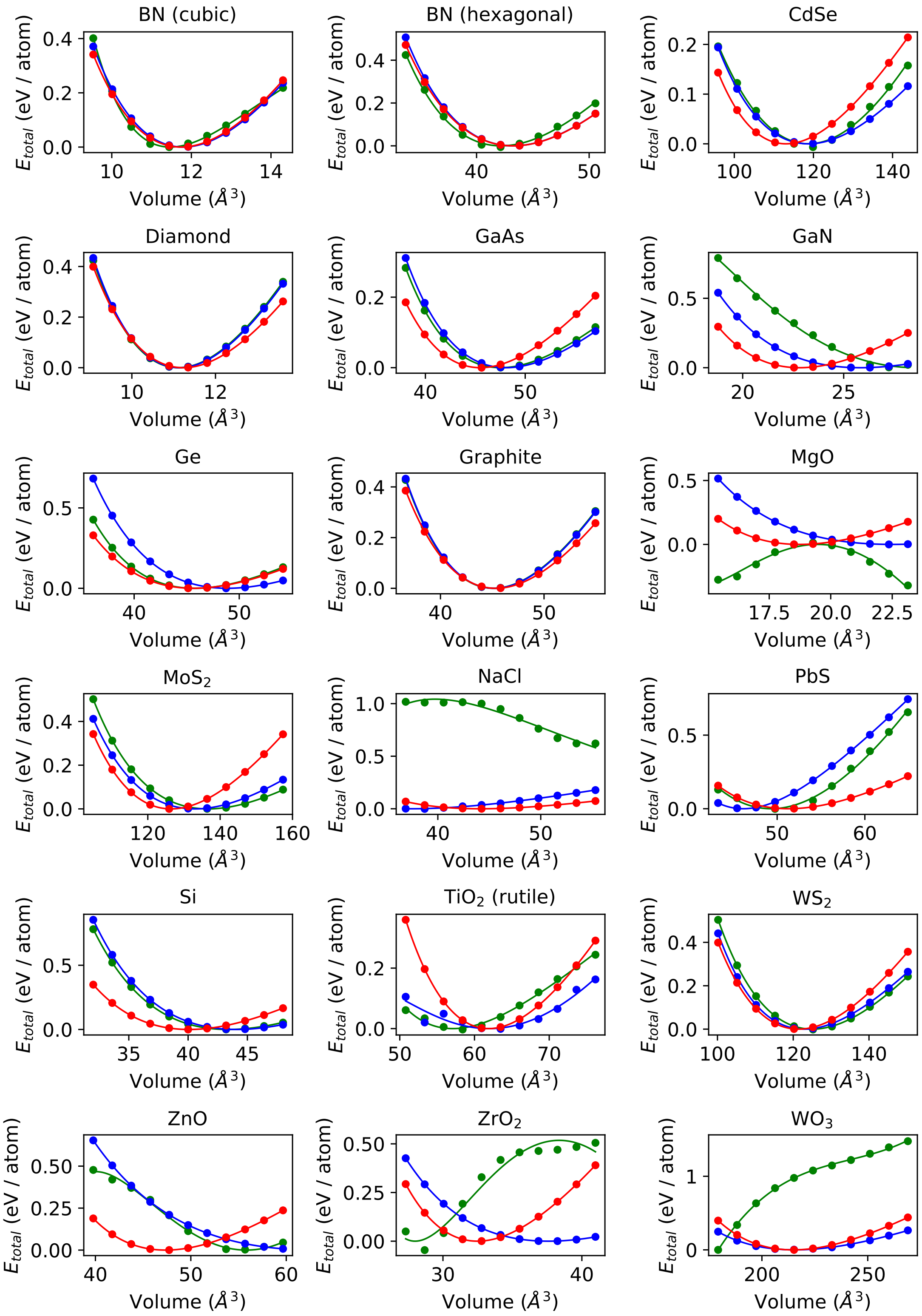

Figure S4: Equation of state curves for the bulk crystal benchmarks, comparing QCore-xTB (blue), GFN1-xTB(s) (green) and DFT (red). In all plots, the x-axis is centered on the DFT equilibrium volume, ranging from $0.9V_0$ to $1.1V_0$, and the y-axis is zeroed at the DFT total energy of the system at V_0.

Table S3: Bulk moduli (in GaP) for the bulk crystal dataset, computed with QCore-xTB, GFN1-xTB(s) and DFT. The relative errors (%) of the tight-binding values w.r.t. DFT are defined as $100|B_{0,TB} - B_{0,DFT}|/B_{0,DFT}$, and are given in parentheses.

System	QCore-xTB	GFN1-xTB(s)	DFT
BN (cubic)	389 (1)	439 (14)	384
BN (hex)	203 (2)	218 (10)	198
CdSe	39 (25)	48 (6)	52
Diamond	530 (18)	532 (19)	448
GaAs	56 (18)	58 (16)	69
GaN	96 (49)	134 (28)	187
Ge	69 (2)	79 (17)	68
Graphite	251 (16)	249 (15)	216
MgO	72 (54)	-319 (303)	157
MoS_2	81 (37)	73 (43)	129
NaCl	4 (86)	17 (33)	26
PbS	154 (159)	113 (90)	59
Si	89 (5)	88 (5)	93
TiO_2 (rutile)	133 (46)	145 (41)	245
WO_3	125 (50)	N/A	250
WS_2	127 (13)	130 (11)	147
ZnO	44 (69)	232 (60)	145
ZrO_2	80 (68)	660 (160)	254
Mean Relative Error (%)	40	51	
Median Relative Error (%)	31	19	

From the definition of density matrix, $\mathbf{D}(\mathbf{k})$, its derivative is:

$$\frac{\partial}{\partial \mathbf{X}}\mathbf{D}(\mathbf{k}) = \frac{\partial}{\partial \mathbf{X}}\mathbf{C}(\mathbf{k})\alpha(\mathbf{k})\mathbf{C}^{\dagger}(\mathbf{k}) + \mathbf{C}(\mathbf{k})\alpha(\mathbf{k})\frac{\partial}{\partial \mathbf{X}}\mathbf{C}^{\dagger}(\mathbf{k}). \tag{25}$$

We also have the normalisation condition:

$$\mathbf{C}^{\dagger}(\mathbf{k})\mathbf{S}(\mathbf{k})\mathbf{C}(\mathbf{k}) = 1, \tag{26}$$

which has the derivative:

$$\begin{aligned} &\left[\frac{\partial}{\partial \mathbf{X}}\mathbf{C}^{\dagger}(\mathbf{k})\right]\mathbf{S}(\mathbf{k})\mathbf{C}(\mathbf{k}) + \mathbf{C}^{\dagger}(\mathbf{k})\mathbf{S}(\mathbf{k})\left[\frac{\partial}{\partial \mathbf{X}}\mathbf{C}(\mathbf{k})\right], \\ &\quad = -\mathbf{C}^{\dagger}(\mathbf{k})\left[\frac{\partial}{\partial \mathbf{X}}\mathbf{S}(\mathbf{k})\right]\mathbf{C}(\mathbf{k}). \end{aligned} \tag{27}$$

Eqs. (25) and (27) allow us to define the trace of the product of the Fock matrix with the derivative of the density matrix as:

$$\begin{aligned} \mathrm{tr}\left[\mathbf{F}(\mathbf{k})\frac{\partial}{\partial \mathbf{X}}\mathbf{D}(\mathbf{k})\bigg|_{\mathbf{F}}\right] &= \mathrm{tr}\left[\mathbf{F}(\mathbf{k})\left(\frac{\partial}{\partial \mathbf{X}}\mathbf{C}(\mathbf{k})\alpha(\mathbf{k})\mathbf{C}^{\dagger}(\mathbf{k})\right.\right. \\ &\qquad \left.\left. +\mathbf{C}(\mathbf{k})\alpha(\mathbf{k})\frac{\partial}{\partial \mathbf{X}}\mathbf{C}^{\dagger}(\mathbf{k})\right)\right] \\ &= \mathrm{tr}\left[\left(\frac{\partial}{\partial \mathbf{X}}\mathbf{C}(\mathbf{k})\right)\alpha(\mathbf{k})\mathbf{C}^{\dagger}(\mathbf{k})\mathbf{F}(\mathbf{k})\right] \\ &\quad + \mathrm{tr}\left[\mathbf{F}(\mathbf{k})\mathbf{C}(\mathbf{k})\alpha(\mathbf{k})\frac{\partial}{\partial \mathbf{X}}\mathbf{C}^{\dagger}(\mathbf{k})\right] \\ &= -\mathrm{tr}\left[\left(\frac{\partial}{\partial \mathbf{X}}S(\mathbf{k})\right)\mathbf{C}(\mathbf{k})\alpha(\mathbf{k})\varepsilon(\mathbf{k})\mathbf{C}^{\dagger}(\mathbf{k})\right], \end{aligned} \tag{28}$$

which shows that the gradient of the density matrix can be defined in terms of the gradient of the overlap matrix, and:

$$\mathbf{C}(\mathbf{k})\alpha(\mathbf{k})\varepsilon(\mathbf{k})\mathbf{C}^{\dagger}(\mathbf{k}), \tag{29}$$

defines the energy-weighted density matrix.

7.0.2 Gradient of the Hamiltonian

The gradient of the core Hamiltonian is given as:

$$
\begin{aligned}
&\frac{\partial}{\partial \mathbf{X}} H^0_{\mu\nu}(\mathbf{T}) \\
&= \frac{1}{2}\left[K_{AB}[1 + k_{EN}\Delta EN^2_{AB}] \frac{\partial h_{\mu\nu}}{\partial \mathbf{R}_C} k^{\text{Hückel}}(\mathbf{T}) S_{\mu\nu}(\mathbf{T}) \Pi(\mathbf{T}) \right. \\
&\quad + K_{AB}[1 + k_{EN}\Delta EN^2_{AB}] h_{\mu\nu} k^{\text{Hückel}}(\mathbf{T}) \frac{\partial S_{\mu\nu}(\mathbf{T})}{\partial \mathbf{X}} \Pi(\mathbf{T}) \\
&\quad \left. + K_{AB}[1 + k_{EN}\Delta EN^2_{AB}] h_{\mu\nu} S_{\mu\nu}(\mathbf{T}) k^{\text{Hückel}}(\mathbf{T}) \frac{\partial \Pi(|\mathbf{R}_{AB} + \mathbf{T}|)}{\partial \mathbf{X}} \right],
\end{aligned} \tag{30}
$$

where:

$$
\frac{\partial h_{Al}}{\partial \mathbf{X}} = H_{Al} k_{CN,l} \frac{\partial CN_A}{\partial \mathbf{R}_C} = \sum_{\mathbf{T}} H_{Al} k_{CN,l} \frac{\partial CN_A(\mathbf{T})}{\partial \mathbf{X}}, \tag{31}
$$

$$
\begin{aligned}
\frac{\partial CN_A(\mathbf{T})}{\partial \mathbf{X}} &= \sum_{B \neq A}^{CN_{\text{cutoff}}} -k_1 \left(\frac{R_{cov,AB}}{|\mathbf{R}_{AB} + \mathbf{T}|^2} \right) e^{-k_1 \left(\frac{R_{cov,AB}}{|\mathbf{R}_{AB}+\mathbf{T}|} \right)} \\
&\quad \times \left[1 + e^{-k_1 \left(\frac{R_{cov,AB}}{|\mathbf{R}_{AB}+\mathbf{T}|} \right)} \right]^{-2} \frac{\partial |\mathbf{R}_{AB} + \mathbf{T}|}{\partial \mathbf{X}},
\end{aligned} \tag{32}
$$

$$
\begin{aligned}
&\frac{\partial \Pi(|\mathbf{R}_{AB} + \mathbf{T}|)}{\partial \mathbf{X}} \\
&= -\left(\frac{1}{2} \frac{k^{\text{poly}}_{A,l} + k^{\text{poly}}_{B,l}}{(R_{cov,AB} |\mathbf{R}_{AB} + \mathbf{T}|)^{1/2}} + \frac{k^{\text{poly}}_{A,l} k^{\text{poly}}_{B,l}}{R_{cov,AB}} \right) \frac{\partial |\mathbf{R}_{AB} + \mathbf{T}|}{\partial \mathbf{X}}.
\end{aligned} \tag{33}
$$

The gradient of the interatomic distance $|\mathbf{R}_{AB} + \mathbf{T}|$ is given as:

$$
\frac{\partial}{\partial \mathbf{R}_C} |\mathbf{R}_{AB} + \mathbf{T}| = \mathbf{n}(\mathbf{R}_{AB} + \mathbf{T}) \delta(A, C) - \mathbf{n}(\mathbf{R}_{AB} + \mathbf{T}) \delta(B, C), \tag{34}
$$

where $\mathbf{n}$ represents the normalized vector, and:

$$
\frac{\partial}{\partial \mathbf{L}} |\mathbf{R}_{AB} + \mathbf{T}| = \mathbf{n}(\mathbf{R}_{AB}) \otimes \mathbf{t}, \text{ where } \mathbf{T}_i \equiv \sum_j \mathbf{L}_{ij} \mathbf{t}_j. \tag{35}
$$

The gradient of the overlap matrix:

$$\frac{\partial \mathbf{S}(\mathbf{k})}{\partial \mathbf{X}} = \sum_{\mathbf{T}} \left(e^{i\mathbf{k}\cdot\mathbf{T}} \frac{\partial S_{\mu\nu}(\mathbf{T})}{\partial \mathbf{X}} + S_{\mu\nu}(\mathbf{T}) \frac{\partial}{\partial \mathbf{X}} e^{i\mathbf{k}\cdot\mathbf{T}} \right). \tag{36}$$

Gradients for the Electrostatic Terms

For the gradient of the second-order electrostatics, we require the derivatives:

$$\frac{\partial p_l^A}{\partial \mathbf{X}} = -\sum_{\mathbf{k}} w^{\mathbf{k}} \sum_{\nu}^{N_{\mathrm{AO}}} \sum_{\mu\in A, \mu\in l} \mathbf{D}_{\nu\mu}(\mathbf{k}) \frac{\partial \mathbf{S}_{\mu\nu}(\mathbf{k})}{\partial \mathbf{X}}, \tag{37}$$

$$\frac{\partial \gamma_{Al,Bl'}}{\partial \mathbf{X}} = \gamma^3_{Al,Bl'} \frac{\partial |\mathbf{R}_{AB} + \mathbf{T}|}{\partial \mathbf{X}}, \tag{38}$$

and:

$$\begin{aligned} \frac{\partial}{\partial \mathbf{R}_C} {}^{2n+1}S_{\mathrm{AB},ll'} = \frac{B(-\frac{1}{2}, n)}{2\Gamma(n+\frac{1}{2})\eta^{2n}_{Al,Bl'}} \Bigg[\\ -\sum_{A\neq B} \Bigg((2n+1) r_{AB}^{-(2n+2)} \Gamma\left(n + \frac{1}{2}, a^2\right) \\ + r_{AB}^{-1} (\pi K^2)^{n+\frac{1}{2}} \exp(-a^2) \Bigg) \frac{\partial |\mathbf{R}_{AB} + \mathbf{T}|}{\partial \mathbf{R}_C} \\ + \frac{2\pi^{2n+1/2}}{V} \sum_{\mathbf{h}_\lambda \neq 0} i\mathbf{h}_\lambda (\delta_{AC} - \delta_{BC}) e^{2\pi i \mathbf{h}_\lambda \cdot \mathbf{R}_{AB}} h_\lambda^{2(n-1)} \Gamma\left(1-n, b^2\right) \Bigg], \end{aligned} \tag{39}$$

$$
\begin{aligned}
\frac{\partial}{\partial \mathbf{L}}\,{}^{2n+1}S_{\mathrm{AB},ll'} = \frac{B(-\frac{1}{2},n)}{2\Gamma(n+\frac{1}{2})\eta_{Al,Bl'}^{2n}}\Bigg[\\
&- \sum_{A\neq B}\Bigg((2n+1)r_{AB}^{-(2n+2)}\Gamma\left(n+\frac{1}{2},a^2\right) \\
&\qquad +r_{AB}^{-1}(\pi K^2)^{n+\frac{1}{2}}\exp(-a^2)\Bigg)\frac{\partial|\mathbf{R}_{AB}+\mathbf{T}|}{\partial \mathbf{L}} \\
&+ \frac{2\pi^{2n+1/2}}{V}\sum_{\mathbf{h}_\lambda\neq 0} i\left(\frac{\partial}{\partial \mathbf{L}}(\mathbf{R}_{AB}\cdot\mathbf{h}_\lambda)\right)e^{2\pi i\mathbf{h}_\lambda\cdot\mathbf{R}_{AB}}h_\lambda^{2(n-1)}\Gamma\left(1-n,b^2\right) \\
&+ \frac{\pi^{2n-1/2}}{V}\sum_{\mathbf{h}_\lambda\neq 0} e^{2\pi i\mathbf{h}_\lambda\cdot\mathbf{R}_{AB}}\Big(2(n-1)h_\lambda^{2n-3}\Gamma(1-n,b^2) \\
&\qquad -\frac{2(\pi K^2)^{1-n}}{h_\lambda}e^{-b^2}\Big)\frac{\partial h_\lambda}{\partial \mathbf{L}} \\
&- \frac{\pi^{2n-1/2}}{V^2}\left(\sum_{\mathbf{h}_\lambda\neq 0} e^{2\pi i\mathbf{h}_\lambda\cdot\mathbf{R}_{AB}}h_\lambda^{2(n-1)}\Gamma(1-n,b^2)+\frac{(\frac{\pi}{K^2})^{1-n}}{n-1}\right)\frac{\partial V}{\partial \mathbf{L}}\Bigg].
\end{aligned} \tag{40}
$$

The gradient of the k-vector,

$$\mathbf{k}_i = \sum_j k_j(\mathbf{L}^{-1})_{ji}, \tag{41}$$

is given as:

$$\frac{\partial \mathbf{k}_i}{\partial \mathbf{L}_{mn}} = -\sum_j \frac{\partial(\mathbf{L}^{-1})_{ji}}{\partial \mathbf{L}_{mn}}n_j = -\sum_j k_j(\mathbf{L}^{-1})_{jm}(\mathbf{L}^{-1})_{ni}, \tag{42}$$

where the gradient of the inverse matrix can be obtained from the following:

$$\frac{\partial}{\partial \mathbf{L}_{mn}}\left[\sum_j \mathbf{L}_{ij}^{-1}\mathbf{L}_{jk}\right] = \frac{\partial}{\partial \mathbf{L}_{mn}}\delta_{ik} = 0.$$

This gives:

$$\left[\frac{\partial}{\partial \mathbf{L}_{mn}}\mathbf{L}_{ij}^{-1}\right]\mathbf{L}_{jk} = -\mathbf{L}_{ij}^{-1}\left[\frac{\partial}{\partial \mathbf{L}_{mn}}\mathbf{L}_{jk}\right] = -\mathbf{L}_{ij}^{-1}\delta_{jm,kn} = -\mathbf{L}_{im}^{-1}\delta_{kn}. \tag{43}$$

Multiplying the inverse matrix of $\mathbf{L}$ on the right for both sides of the equation gives:

$$\begin{aligned}\left[\frac{\partial}{\partial \mathbf{L}_{mn}}\mathbf{L}^{-1}_{ij}\right]\mathbf{L}_{jk}\mathbf{L}^{-1}_{kl} = \left[\frac{\partial}{\partial \mathbf{L}_{mn}}\mathbf{L}^{-1}_{ij}\right]\delta_{jl} &= \left[\frac{\partial}{\partial \mathbf{L}_{mn}}\mathbf{L}^{-1}_{il}\right], \\ &= -\mathbf{L}^{-1}_{im}\delta_{kn}\mathbf{L}^{-1}_{kl}, \\ &= -\mathbf{L}^{-1}_{im}\mathbf{L}^{-1}_{nl},\end{aligned} \tag{44}$$

which results in:

$$\frac{\partial}{\partial \mathbf{L}_{mn}}\mathbf{L}^{-1}_{il} = -\mathbf{L}^{-1}_{im}\mathbf{L}^{-1}_{nl}. \tag{45}$$

We also have:

$$\left(\frac{\partial}{\partial \mathbf{L}}(\mathbf{R}_{AB}\cdot\mathbf{k})\right)_{mn} = \sum_i\sum_j k_j(\mathbf{L}^{-1})_{jm}(\mathbf{L}^{-1})_{ni}(\mathbf{R}_{AB})_i, \tag{46}$$

and:

$$\frac{1}{V}\frac{\partial V}{\partial \mathbf{L}} = \mathbf{L}^{-1}. \tag{47}$$

For the third-order electrostatic term, the gradient is simply:

$$\frac{\partial}{\partial \mathbf{X}}H_3 = \sum_A \Gamma_A q_A^2\frac{\partial q_A}{\partial \mathbf{X}} = \sum_A \Gamma_A q_A^2 \sum_{l\in A}\frac{\partial p_l^A}{\partial \mathbf{X}}. \tag{48}$$

References

(S1) Grimme, S.; Antony, J.; Ehrlich, S.; Krieg, H. A Consistent and Accurate Ab Initio Parametrization of Density Functional Dispersion Correction (DFT-F) for the 94 Elements H-Pu. *The Journal of Chemical Physics* **2010**, *132*.

(S2) Grimme, S.; Bannwarth, C.; Shushkov, P. A Robust and Accurate Tight-Binding Quantum Chemical Method for Structures, Vibrational Frequencies, and Noncovalent Interactions of Large Molecular Systems Parametrized for All spd-block Elements (Z = 1–86). *Journal of Chemical Theory and Computation* **2017**, *13*, 1989–2009.

(S3) Kühne, T. D.; Iannuzzi, M.; Del Ben, M.; Rybkin, V. V.; Seewald, P.; Stein, F.;

Laino, T.; Khaliullin, R. Z.; Schütt, O.; Schiffmann, F., et al. CP2K: An Electronic Structure and Molecular Dynamics Software Package-Quickstep: Efficient and Accurate Electronic Structure Calculations. *The Journal of Chemical Physics* **2020**, *152*, 194103.

(S4) Ehlert, S. Tblite. `https://github.com/tblite/tblite`, 2022; Version tag: 0.2.1.

(S5) Williams, D. E. Accelerated Convergence of Crystal-Lattice Potential Sums. *Acta Crystallographica Section A: Crystal Physics, Diffraction, Theoretical and General Crystallography* **1971**, *27*, 452–455.

(S6) Olver, F. NIST Handbook of Mathematical Functions. *US Department of Commerce, National Institute of Standards and Technology* **2010**,

(S7) Bracewell, R.; Kahn, P. B. The Fourier Transform and its Applications. *American Journal of Physics* **1966**, *34*, 712–712.

(S8) Inc., W. R. Mathematica, version 13.3. `https://www.wolfram.com/mathematica`, Champaign, IL, 2023.

(S9) Jain, A.; Ong, S. P.; Hautier, G.; Chen, W.; Richards, W. D.; Dacek, S.; Cholia, S.; Gunter, D.; Skinner, D.; Ceder, G., et al. Commentary: The Materials Project: A Materials Genome Approach to Accelerating Materials Innovation. *APL Materials* **2013**, *1*.

(S10) Yin, M.; Cohen, M. L. Ground-state Properties of Diamond. *Physical Review B* **1981**, *24*, 6121.

(S11) Larsen, A. H.; Mortensen, J. J.; Blomqvist, J.; Castelli, I. E.; Christensen, R.; Dułak, M.; Friis, J.; Groves, M. N.; Hammer, B.; Hargus, C., et al. The Atomic Simulation Environment–a Python Library for Working with Atoms. *Journal of Physics: Condensed Matter* **2017**, *29*, 273002.

(S12) Buccheri, A.; Li, R. Periodicxtbworkflows. `https://github.com/AlexBuccheri/periodicXTBworkflows`, 2023-2024; Version: v1.0.0.

(S13) Monkhorst, H. J.; Pack, J. D. Special Points for Brillouin-Zone Integrations. *Physical Review B* **1976**, *13*, 5188–5192.

(S14) Reilly, A. M.; Tkatchenko, A. Understanding the Role of Vibrations, Exact Exchange, and Many-Body Van der Waals Interactions in the Cohesive Properties of Molecular Crystals. *The Journal of Chemical Physics* **2013**, *139*, 024705.

(S15) Birch, F. Finite Elastic Strain of Cubic Crystals. *Physical Review* **1947**, *71*, 809.
